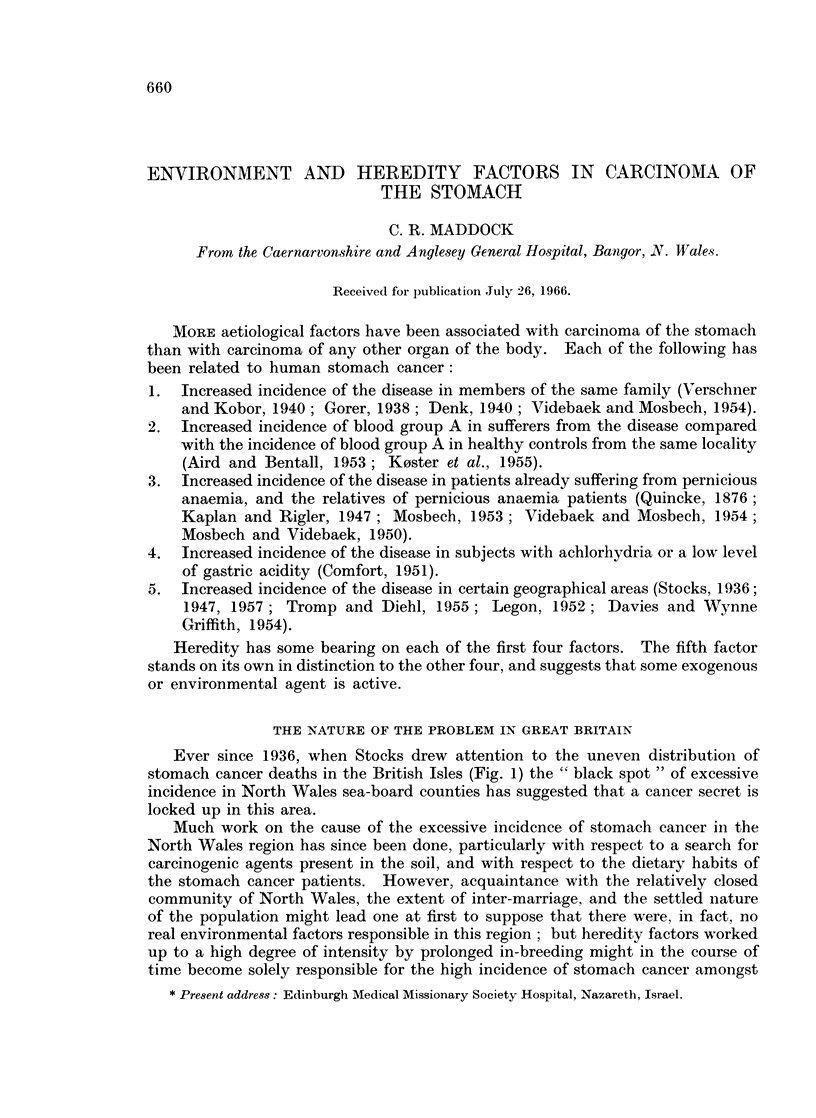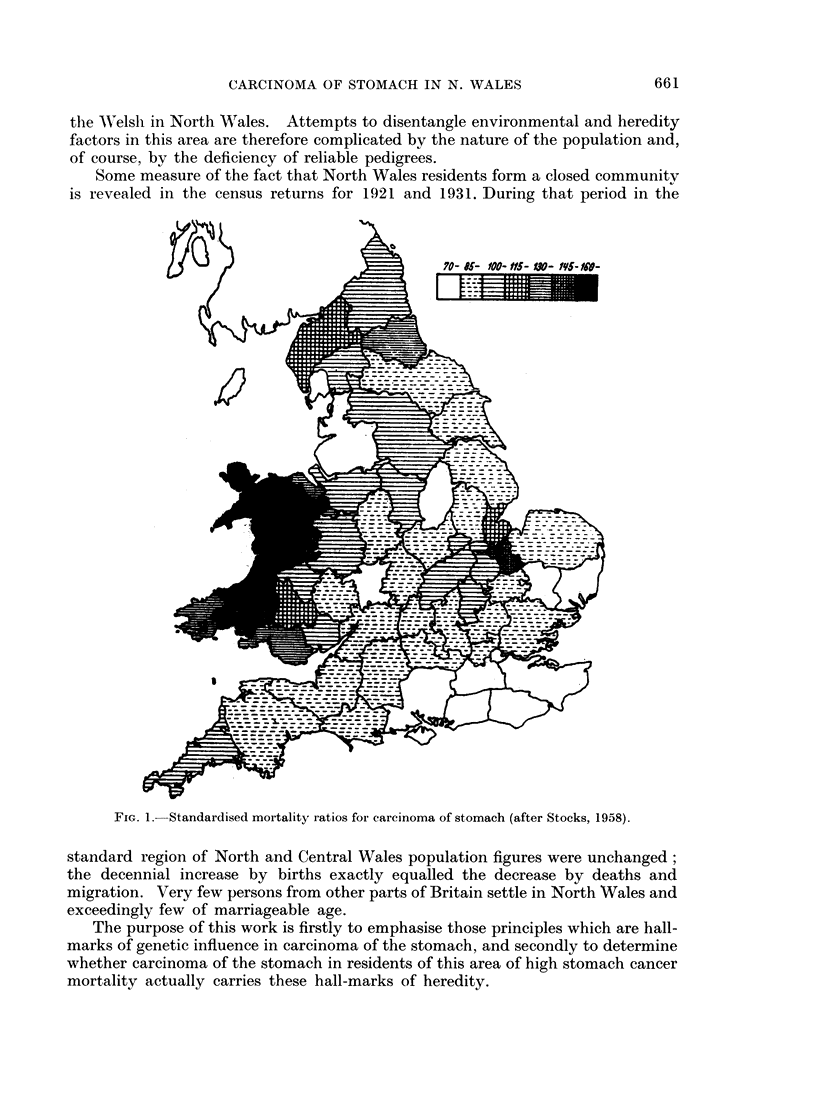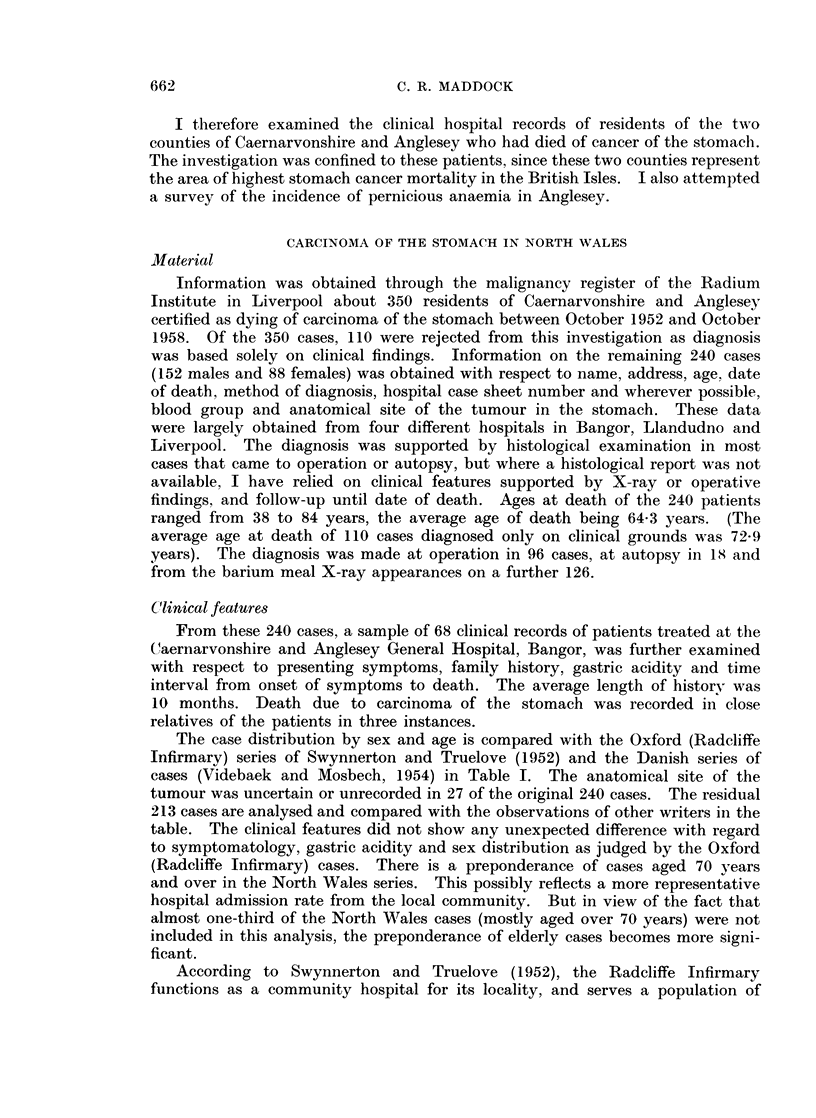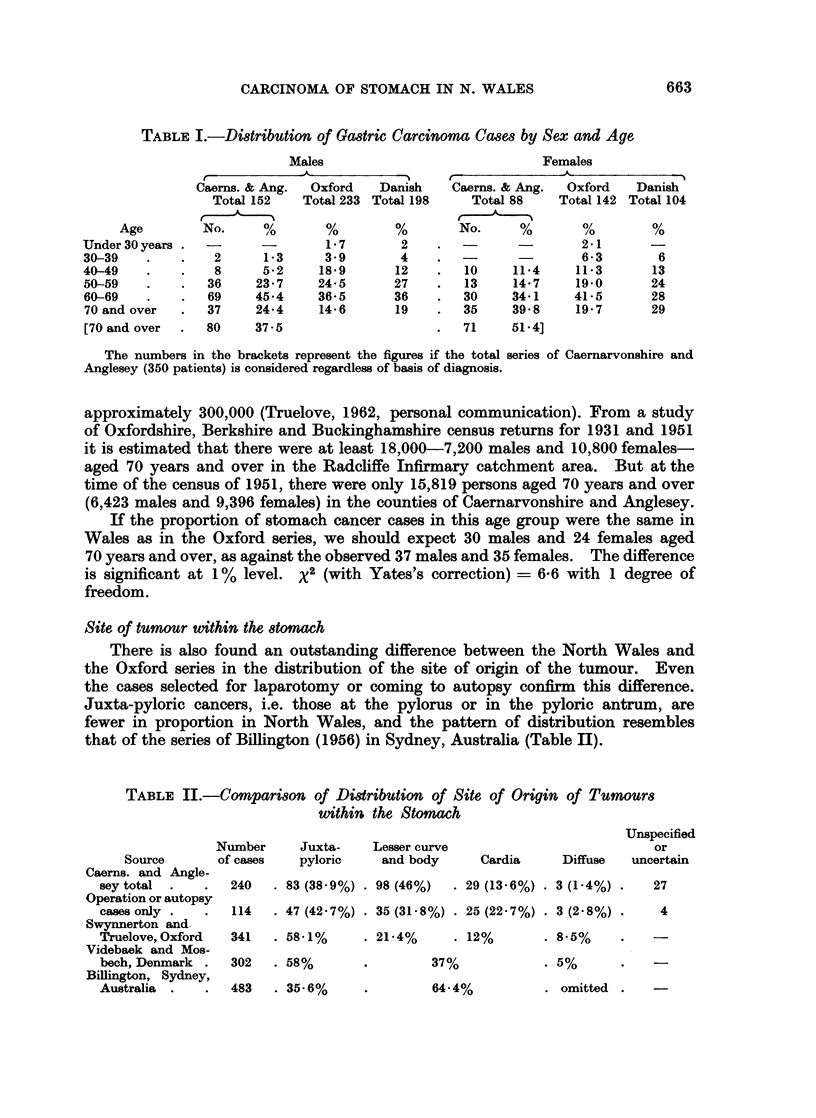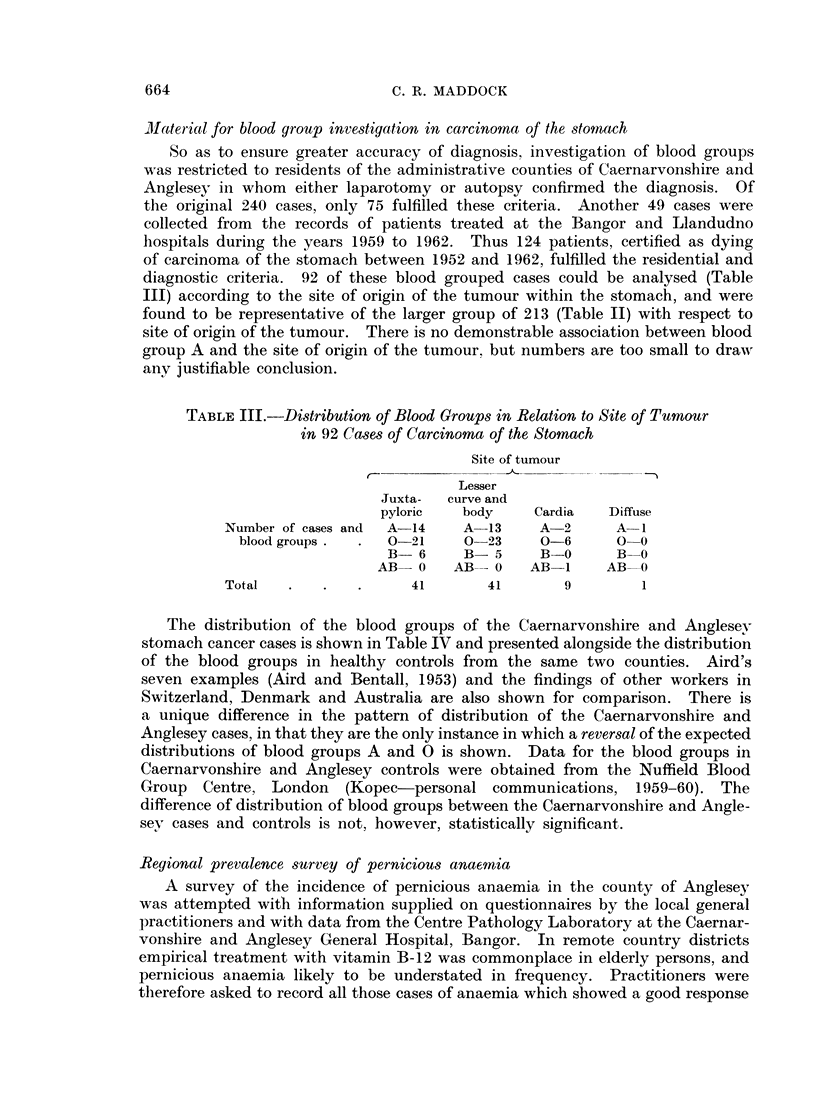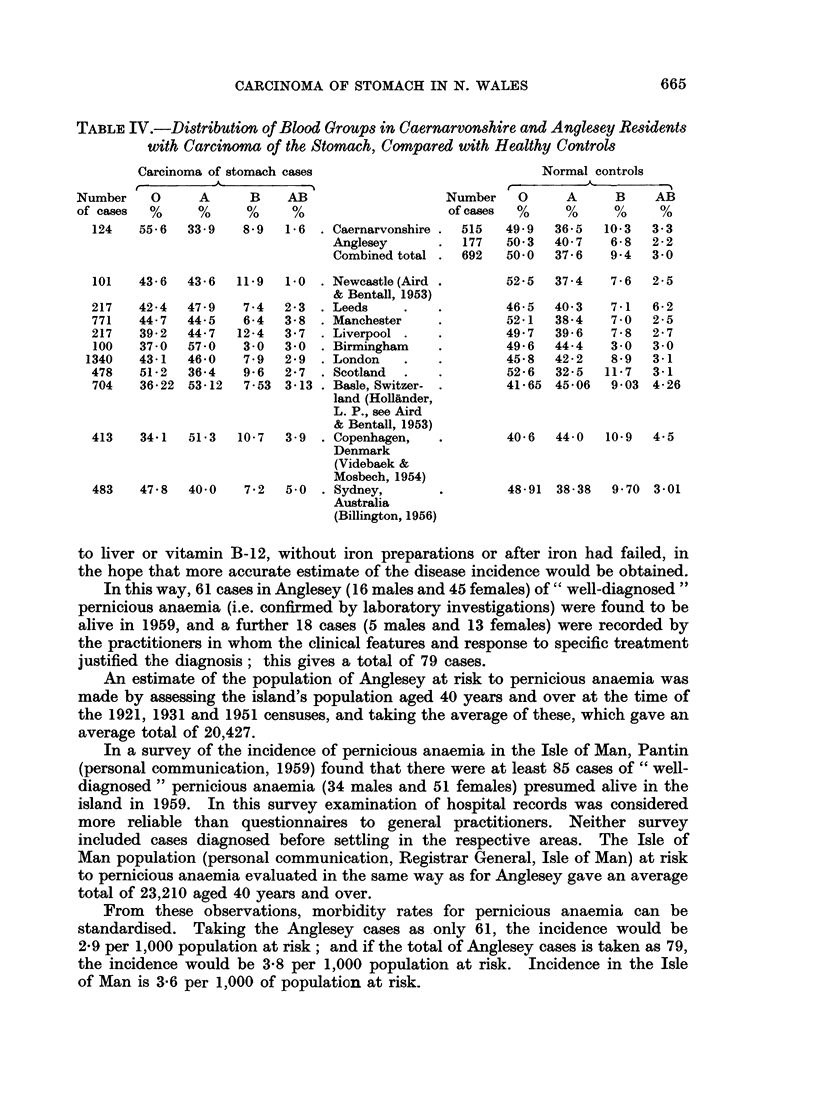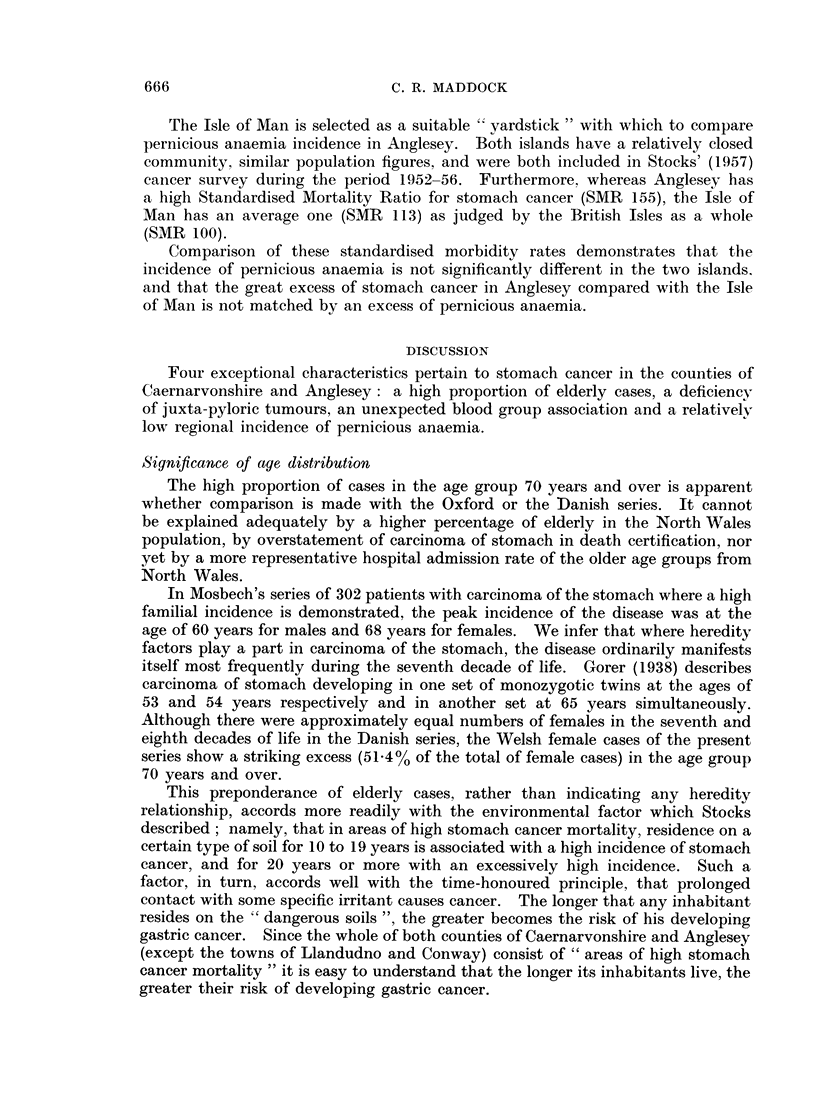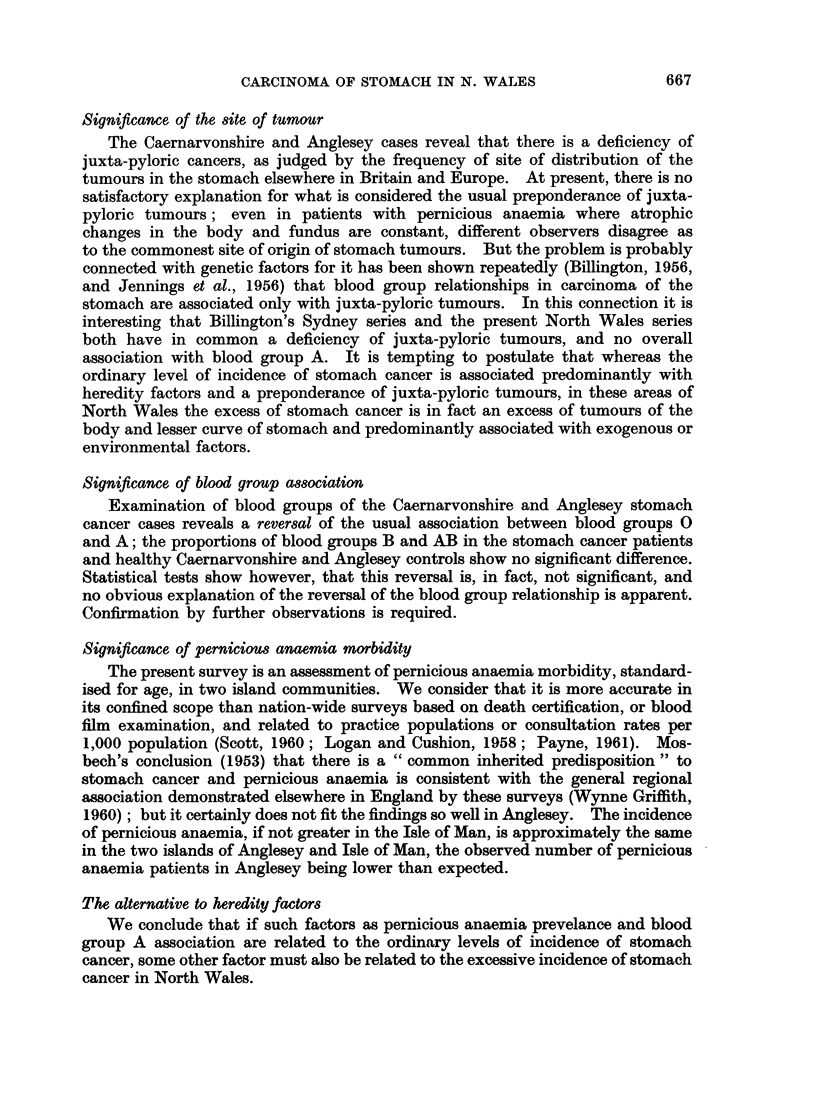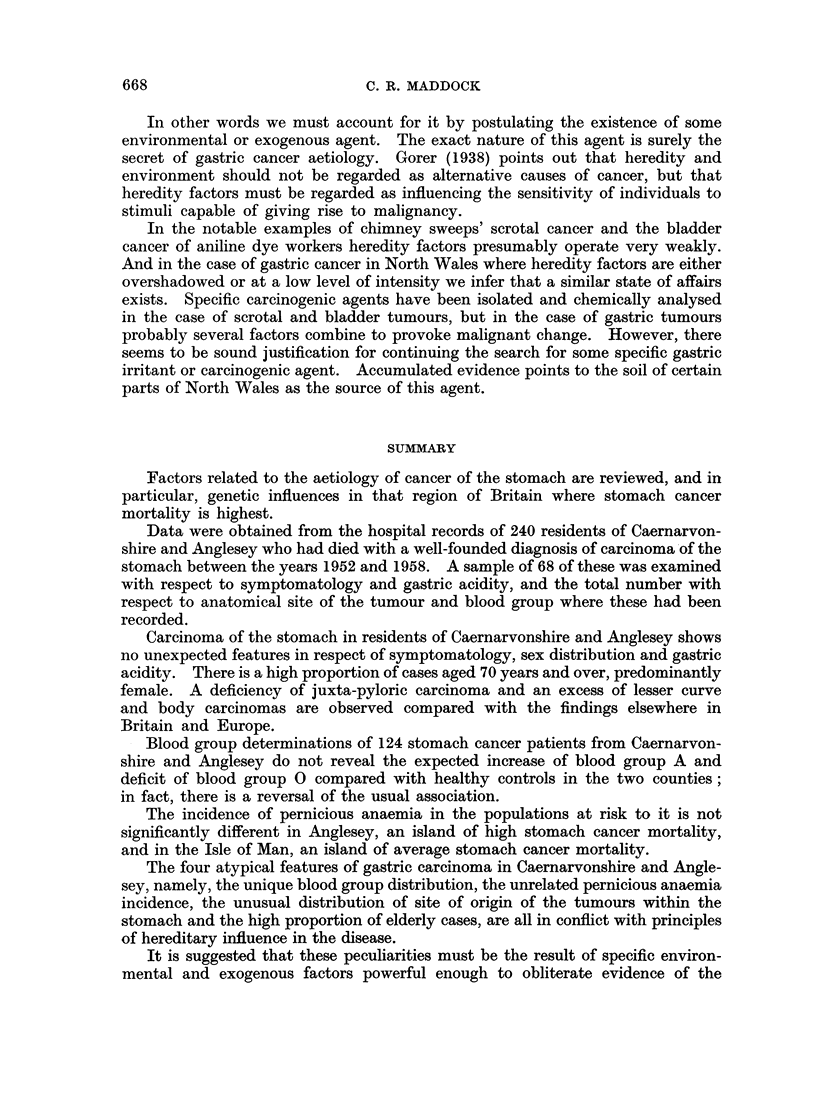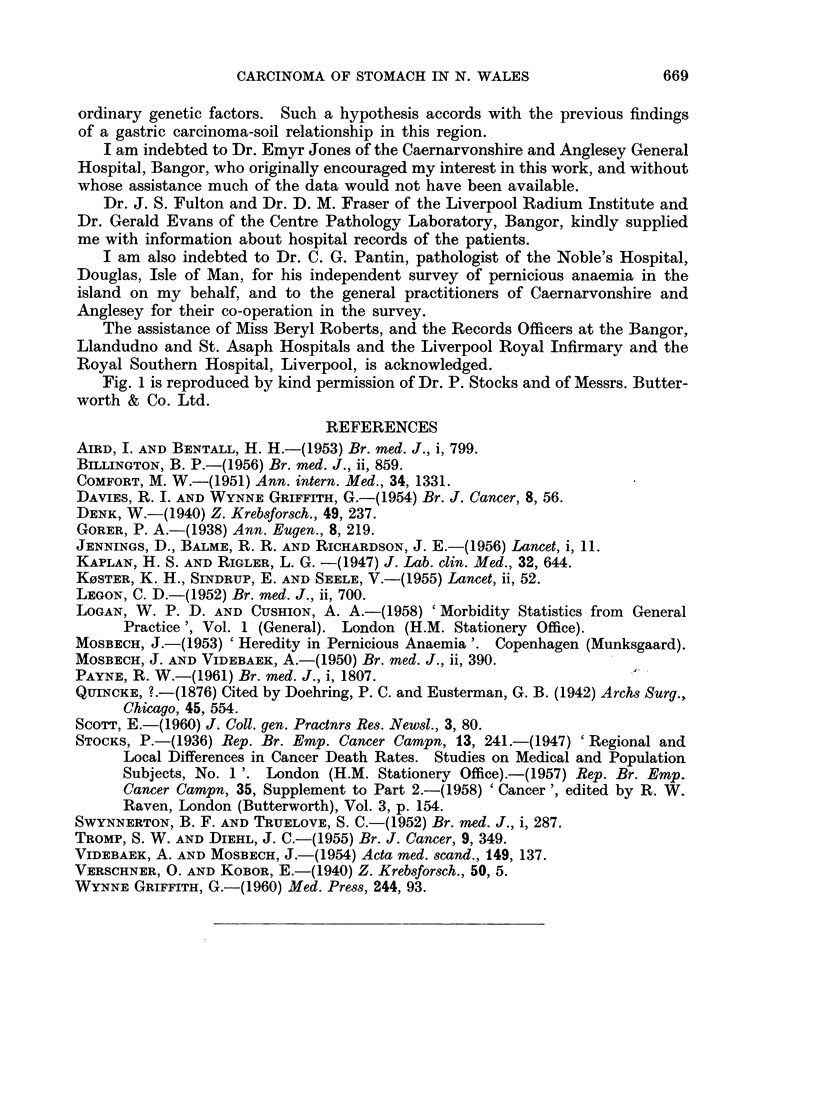# Environment and heredity factors in carcinoma of the stomach.

**DOI:** 10.1038/bjc.1966.77

**Published:** 1966-12

**Authors:** C. R. Maddock


### ENVIRONMENT AND HEREDITY FACTORS IN CARCINOMA OF THE STOMACH

C. R. MADDOCK

*From the Caernarvonshire and Anglesey General Hospital, Bangor, N. Wales.*

Received for publication July 26, 1966.

MORE aetiological factors have been associated with carcinoma of the stomach than with carcinoma of any other organ of the body. Each of the following has been related to human stomach cancer :

1. Increased incidence of the disease in members of the same family (Verschner and Kobor, 1940 ; Gorer, 1938 ; Denk, 1940 ; Videbaek and Mosbech, 1954).
2. Increased incidence of blood group A in sufferers from the disease compared with the incidence of blood group A in healthy controls from the same locality (Aird and Bentall, 1953 ; Køster *et al.*, 1955).
3. Increased incidence of the disease in patients already suffering from pernicious anaemia, and the relatives of pernicious anaemia patients (Quincke, 1876 ; Kaplan and Rigler, 1947 ; Mosbech, 1953 ; Videbaek and Mosbech, 1954 ; Mosbech and Videbaek, 1950).
4. Increased incidence of the disease in subjects with achlorhydria or a low level of gastric acidity (Comfort, 1951).
5. Increased incidence of the disease in certain geographical areas (Stocks, 1936 ; 1947, 1957 ; Tromp and Diehl, 1955 ; Legon, 1952 ; Davies and Wynne Griffith, 1954).

Heredity has some bearing on each of the first four factors. The fifth factor stands on its own in distinction to the other four, and suggests that some exogenous or environmental agent is active.

### THE NATURE OF THE PROBLEM IN GREAT BRITAIN

Ever since 1936, when Stocks drew attention to the uneven distribution of stomach cancer deaths in the British Isles (Fig. 1) the " black spot " of excessive incidence in North Wales sea-board counties has suggested that a cancer secret is locked up in this area.

Much work on the cause of the excessive incidence of stomach cancer in the North Wales region has since been done, particularly with respect to a search for carcinogenic agents present in the soil, and with respect to the dietary habits of the stomach cancer patients. However, acquaintance with the relatively closed community of North Wales, the extent of inter-marriage, and the settled nature of the population might lead one at first to suppose that there were, in fact, no real environmental factors responsible in this region ; but heredity factors worked up to a high degree of intensity by prolonged in-breeding might in the course of time become solely responsible for the high incidence of stomach cancer amongst

* *Present address :* Edinburgh Medical Missionary Society Hospital, Nazareth, Israel.

the Welsh in North Wales. Attempts to disentangle environmental and heredity factors in this area are therefore complicated by the nature of the population and, of course, by the deficiency of reliable pedigrees.

Some measure of the fact that North Wales residents form a closed community is revealed in the census returns for 1921 and 1931. During that period in the standard region of North and Central Wales population figures were unchanged ; the decennial increase by births exactly equalled the decrease by deaths and migration. Very few persons from other parts of Britain settle in North Wales and exceedingly few of marriageable age.

FIG. 1.—Standardised mortality ratios for carcinoma of stomach (after Stocks, 1958).

The purpose of this work is firstly to emphasise those principles which are hall-marks of genetic influence in carcinoma of the stomach, and secondly to determine whether carcinoma of the stomach in residents of this area of high stomach cancer mortality actually carries these hall-marks of heredity.

I therefore examined the clinical hospital records of residents of the two counties of Caernarvonshire and Anglesey who had died of cancer of the stomach. The investigation was confined to these patients, since these two counties represent the area of highest stomach cancer mortality in the British Isles. I also attempted a survey of the incidence of pernicious anaemia in Anglesey.

### CARCINOMA OF THE STOMACH IN NORTH WALES

### *Material*

Information was obtained through the malignancy register of the Radium Institute in Liverpool about 350 residents of Caernarvonshire and Anglesey certified as dying of carcinoma of the stomach between October 1952 and October 1958. Of the 350 cases, 110 were rejected from this investigation as diagnosis was based solely on clinical findings. Information on the remaining 240 cases (152 males and 88 females) was obtained with respect to name, address, age, date of death, method of diagnosis, hospital case sheet number and wherever possible, blood group and anatomical site of the tumour in the stomach. These data were largely obtained from four different hospitals in Bangor, Llandudno and Liverpool. The diagnosis was supported by histological examination in most cases that came to operation or autopsy, but where a histological report was not available, I have relied on clinical features supported by X-ray or operative findings, and follow-up until date of death. Ages at death of the 240 patients ranged from 38 to 84 years, the average age of death being 64·3 years. (The average age at death of 110 cases diagnosed only on clinical grounds was 72·9 years). The diagnosis was made at operation in 96 cases, at autopsy in 18 and from the barium meal X-ray appearances on a further 126.

### *Clinical features*

From these 240 cases, a sample of 68 clinical records of patients treated at the Caernarvonshire and Anglesey General Hospital, Bangor, was further examined with respect to presenting symptoms, family history, gastric acidity and time interval from onset of symptoms to death. The average length of history was 10 months. Death due to carcinoma of the stomach was recorded in close relatives of the patients in three instances.

The case distribution by sex and age is compared with the Oxford (Radcliffe Infirmary) series of Swynnerton and Truelove (1952) and the Danish series of cases (Videbaek and Mosbech, 1954) in Table I. The anatomical site of the tumour was uncertain or unrecorded in 27 of the original 240 cases. The residual 213 cases are analysed and compared with the observations of other writers in the table. The clinical features did not show any unexpected difference with regard to symptomatology, gastric acidity and sex distribution as judged by the Oxford (Radcliffe Infirmary) cases. There is a preponderance of cases aged 70 years and over in the North Wales series. This possibly reflects a more representative hospital admission rate from the local community. But in view of the fact that almost one-third of the North Wales cases (mostly aged over 70 years) were not included in this analysis, the preponderance of elderly cases becomes more significant.

According to Swynnerton and Truelove (1952), the Radcliffe Infirmary functions as a community hospital for its locality, and serves a population of

TABLE I.—*Distribution of Gastric Carcinoma Cases by Sex and Age*

| | Males | | | | Females | | | |
|---|---|---|---|---|---|---|---|---|
| | Caerns. & Ang. Total 152 | | Oxford Total 233 | Danish Total 198 | Caerns. & Ang. Total 88 | | Oxford Total 142 | Danish Total 104 |
| Age | No. | % | % | % | No. | % | % | % |
| Under 30 years | — | — | 1·7 | 2 | — | — | 2·1 | — |
| 30–39 | 2 | 1·3 | 3·9 | 4 | — | — | 6·3 | 6 |
| 40–49 | 8 | 5·2 | 18·9 | 12 | 10 | 11·4 | 11·3 | 13 |
| 50–59 | 36 | 23·7 | 24·5 | 27 | 13 | 14·7 | 19·0 | 24 |
| 60–69 | 69 | 45·4 | 36·5 | 36 | 30 | 34·1 | 41·5 | 28 |
| 70 and over | 37 | 24·4 | 14·6 | 19 | 35 | 39·8 | 19·7 | 29 |
| [70 and over | 80 | 37·5 | | | 71 | 51·4] | | |

The numbers in the brackets represent the figures if the total series of Caernarvonshire and Anglesey (350 patients) is considered regardless of basis of diagnosis.

approximately 300,000 (Truelove, 1962, personal communication). From a study of Oxfordshire, Berkshire and Buckinghamshire census returns for 1931 and 1951 it is estimated that there were at least 18,000—7,200 males and 10,800 females—aged 70 years and over in the Radcliffe Infirmary catchment area. But at the time of the census of 1951, there were only 15,819 persons aged 70 years and over (6,423 males and 9,396 females) in the counties of Caernarvonshire and Anglesey.

If the proportion of stomach cancer cases in this age group were the same in Wales as in the Oxford series, we should expect 30 males and 24 females aged 70 years and over, as against the observed 37 males and 35 females. The difference is significant at 1% level. $\chi^2$ (with Yates's correction) = 6·6 with 1 degree of freedom.

*Site of tumour within the stomach*

There is also found an outstanding difference between the North Wales and the Oxford series in the distribution of the site of origin of the tumour. Even the cases selected for laparotomy or coming to autopsy confirm this difference. Juxta-pyloric cancers, i.e. those at the pylorus or in the pyloric antrum, are fewer in proportion in North Wales, and the pattern of distribution resembles that of the series of Billington (1956) in Sydney, Australia (Table II).

TABLE II.—*Comparison of Distribution of Site of Origin of Tumours within the Stomach*

| Source | Number of cases | Juxta-pyloric | Lesser curve and body | Cardia | Diffuse | Unspecified or uncertain |
|---|---|---|---|---|---|---|
| Caerns. and Anglesey total | 240 | 83 (38·9%) | 98 (46%) | 29 (13·6%) | 3 (1·4%) | 27 |
| Operation or autopsy cases only | 114 | 47 (42·7%) | 35 (31·8%) | 25 (22·7%) | 3 (2·8%) | 4 |
| Swynnerton and Truelove, Oxford | 341 | 58·1% | 21·4% | 12% | 8·5% | — |
| Videbaek and Mosbech, Denmark | 302 | 58% | 37% | | 5% | — |
| Billington, Sydney, Australia | 483 | 35·6% | 64·4% | | omitted | — |

*Material for blood group investigation in carcinoma of the stomach*

So as to ensure greater accuracy of diagnosis, investigation of blood groups was restricted to residents of the administrative counties of Caernarvonshire and Anglesey in whom either laparotomy or autopsy confirmed the diagnosis. Of the original 240 cases, only 75 fulfilled these criteria. Another 49 cases were collected from the records of patients treated at the Bangor and Llandudno hospitals during the years 1959 to 1962. Thus 124 patients, certified as dying of carcinoma of the stomach between 1952 and 1962, fulfilled the residential and diagnostic criteria. 92 of these blood grouped cases could be analysed (Table III) according to the site of origin of the tumour within the stomach, and were found to be representative of the larger group of 213 (Table II) with respect to site of origin of the tumour. There is no demonstrable association between blood group A and the site of origin of the tumour, but numbers are too small to draw any justifiable conclusion.

TABLE III.—*Distribution of Blood Groups in Relation to Site of Tumour in 92 Cases of Carcinoma of the Stomach*

| | Site of tumour | | | |
|---|---|---|---|---|
| | Juxta-pyloric | Lesser curve and body | Cardia | Diffuse |
| Number of cases and blood groups . . | A—14 | A—13 | A—2 | A—1 |
| | O—21 | O—23 | O—6 | O—0 |
| | B— 6 | B— 5 | B—0 | B—0 |
| | AB— 0 | AB— 0 | AB—1 | AB—0 |
| Total . . . | 41 | 41 | 9 | 1 |

The distribution of the blood groups of the Caernarvonshire and Anglesey stomach cancer cases is shown in Table IV and presented alongside the distribution of the blood groups in healthy controls from the same two counties. Aird's seven examples (Aird and Bentall, 1953) and the findings of other workers in Switzerland, Denmark and Australia are also shown for comparison. There is a unique difference in the pattern of distribution of the Caernarvonshire and Anglesey cases, in that they are the only instance in which a *reversal* of the expected distributions of blood groups A and O is shown. Data for the blood groups in Caernarvonshire and Anglesey controls were obtained from the Nuffield Blood Group Centre, London (Kopec—personal communications, 1959–60). The difference of distribution of blood groups between the Caernarvonshire and Anglesey cases and controls is not, however, statistically significant.

*Regional prevalence survey of pernicious anaemia*

A survey of the incidence of pernicious anaemia in the county of Anglesey was attempted with information supplied on questionnaires by the local general practitioners and with data from the Centre Pathology Laboratory at the Caernarvonshire and Anglesey General Hospital, Bangor. In remote country districts empirical treatment with vitamin B-12 was commonplace in elderly persons, and pernicious anaemia likely to be understated in frequency. Practitioners were therefore asked to record all those cases of anaemia which showed a good response

TABLE IV.—*Distribution of Blood Groups in Caernarvonshire and Anglesey Residents with Carcinoma of the Stomach, Compared with Healthy Controls*

| Carcinoma of stomach cases | | | | | | Normal controls | | | | |
|---|---|---|---|---|---|---|---|---|---|---|
| Number of cases | O % | A % | B % | AB % | | Number of cases | O % | A % | B % | AB % |
| 124 | 55·6 | 33·9 | 8·9 | 1·6 | Caernarvonshire | 515 | 49·9 | 36·5 | 10·3 | 3·3 |
| | | | | | Anglesey | 177 | 50·3 | 40·7 | 6·8 | 2·2 |
| | | | | | Combined total | 692 | 50·0 | 37·6 | 9·4 | 3·0 |
| 101 | 43·6 | 43·6 | 11·9 | 1·0 | Newcastle (Aird & Bentall, 1953) | | 52·5 | 37·4 | 7·6 | 2·5 |
| 217 | 42·4 | 47·9 | 7·4 | 2·3 | Leeds | | 46·5 | 40·3 | 7·1 | 6·2 |
| 771 | 44·7 | 44·5 | 6·4 | 3·8 | Manchester | | 52·1 | 38·4 | 7·0 | 2·5 |
| 217 | 39·2 | 44·7 | 12·4 | 3·7 | Liverpool | | 49·7 | 39·6 | 7·8 | 2·7 |
| 100 | 37·0 | 57·0 | 3·0 | 3·0 | Birmingham | | 49·6 | 44·4 | 3·0 | 3·0 |
| 1340 | 43·1 | 46·0 | 7·9 | 2·9 | London | | 45·8 | 42·2 | 8·9 | 3·1 |
| 478 | 51·2 | 36·4 | 9·6 | 2·7 | Scotland | | 52·6 | 32·5 | 11·7 | 3·1 |
| 704 | 36·22 | 53·12 | 7·53 | 3·13 | Basle, Switzerland (Holländer, L. P., see Aird & Bentall, 1953) | | 41·65 | 45·06 | 9·03 | 4·26 |
| 413 | 34·1 | 51·3 | 10·7 | 3·9 | Copenhagen, Denmark (Videbaek & Mosbech, 1954) | | 40·6 | 44·0 | 10·9 | 4·5 |
| 483 | 47·8 | 40·0 | 7·2 | 5·0 | Sydney, Australia (Billington, 1956) | | 48·91 | 38·38 | 9·70 | 3·01 |

to liver or vitamin B-12, without iron preparations or after iron had failed, in the hope that more accurate estimate of the disease incidence would be obtained.

In this way, 61 cases in Anglesey (16 males and 45 females) of " well-diagnosed " pernicious anaemia (i.e. confirmed by laboratory investigations) were found to be alive in 1959, and a further 18 cases (5 males and 13 females) were recorded by the practitioners in whom the clinical features and response to specific treatment justified the diagnosis ; this gives a total of 79 cases.

An estimate of the population of Anglesey at risk to pernicious anaemia was made by assessing the island's population aged 40 years and over at the time of the 1921, 1931 and 1951 censuses, and taking the average of these, which gave an average total of 20,427.

In a survey of the incidence of pernicious anaemia in the Isle of Man, Pantin (personal communication, 1959) found that there were at least 85 cases of " well-diagnosed " pernicious anaemia (34 males and 51 females) presumed alive in the island in 1959. In this survey examination of hospital records was considered more reliable than questionnaires to general practitioners. Neither survey included cases diagnosed before settling in the respective areas. The Isle of Man population (personal communication, Registrar General, Isle of Man) at risk to pernicious anaemia evaluated in the same way as for Anglesey gave an average total of 23,210 aged 40 years and over.

From these observations, morbidity rates for pernicious anaemia can be standardised. Taking the Anglesey cases as only 61, the incidence would be 2·9 per 1,000 population at risk ; and if the total of Anglesey cases is taken as 79, the incidence would be 3·8 per 1,000 population at risk. Incidence in the Isle of Man is 3·6 per 1,000 of population at risk.

The Isle of Man is selected as a suitable " yardstick " with which to compare pernicious anaemia incidence in Anglesey. Both islands have a relatively closed community, similar population figures, and were both included in Stocks' (1957) cancer survey during the period 1952–56. Furthermore, whereas Anglesey has a high Standardised Mortality Ratio for stomach cancer (SMR 155), the Isle of Man has an average one (SMR 113) as judged by the British Isles as a whole (SMR 100).

Comparison of these standardised morbidity rates demonstrates that the incidence of pernicious anaemia is not significantly different in the two islands, and that the great excess of stomach cancer in Anglesey compared with the Isle of Man is not matched by an excess of pernicious anaemia.

### DISCUSSION

Four exceptional characteristics pertain to stomach cancer in the counties of Caernarvonshire and Anglesey : a high proportion of elderly cases, a deficiency of juxta-pyloric tumours, an unexpected blood group association and a relatively low regional incidence of pernicious anaemia.

### *Significance of age distribution*

The high proportion of cases in the age group 70 years and over is apparent whether comparison is made with the Oxford or the Danish series. It cannot be explained adequately by a higher percentage of elderly in the North Wales population, by overstatement of carcinoma of stomach in death certification, nor yet by a more representative hospital admission rate of the older age groups from North Wales.

In Mosbech's series of 302 patients with carcinoma of the stomach where a high familial incidence is demonstrated, the peak incidence of the disease was at the age of 60 years for males and 68 years for females. We infer that where heredity factors play a part in carcinoma of the stomach, the disease ordinarily manifests itself most frequently during the seventh decade of life. Gorer (1938) describes carcinoma of stomach developing in one set of monozygotic twins at the ages of 53 and 54 years respectively and in another set at 65 years simultaneously. Although there were approximately equal numbers of females in the seventh and eighth decades of life in the Danish series, the Welsh female cases of the present series show a striking excess (51·4% of the total of female cases) in the age group 70 years and over.

This preponderance of elderly cases, rather than indicating any heredity relationship, accords more readily with the environmental factor which Stocks described ; namely, that in areas of high stomach cancer mortality, residence on a certain type of soil for 10 to 19 years is associated with a high incidence of stomach cancer, and for 20 years or more with an excessively high incidence. Such a factor, in turn, accords well with the time-honoured principle, that prolonged contact with some specific irritant causes cancer. The longer that any inhabitant resides on the " dangerous soils ", the greater becomes the risk of his developing gastric cancer. Since the whole of both counties of Caernarvonshire and Anglesey (except the towns of Llandudno and Conway) consist of " areas of high stomach cancer mortality " it is easy to understand that the longer its inhabitants live, the greater their risk of developing gastric cancer.

*Significance of the site of tumour*

The Caernarvonshire and Anglesey cases reveal that there is a deficiency of juxta-pyloric cancers, as judged by the frequency of site of distribution of the tumours in the stomach elsewhere in Britain and Europe. At present, there is no satisfactory explanation for what is considered the usual preponderance of juxta-pyloric tumours; even in patients with pernicious anaemia where atrophic changes in the body and fundus are constant, different observers disagree as to the commonest site of origin of stomach tumours. But the problem is probably connected with genetic factors for it has been shown repeatedly (Billington, 1956, and Jennings *et al.*, 1956) that blood group relationships in carcinoma of the stomach are associated only with juxta-pyloric tumours. In this connection it is interesting that Billington's Sydney series and the present North Wales series both have in common a deficiency of juxta-pyloric tumours, and no overall association with blood group A. It is tempting to postulate that whereas the ordinary level of incidence of stomach cancer is associated predominantly with heredity factors and a preponderance of juxta-pyloric tumours, in these areas of North Wales the excess of stomach cancer is in fact an excess of tumours of the body and lesser curve of stomach and predominantly associated with exogenous or environmental factors.

*Significance of blood group association*

Examination of blood groups of the Caernarvonshire and Anglesey stomach cancer cases reveals a *reversal* of the usual association between blood groups O and A; the proportions of blood groups B and AB in the stomach cancer patients and healthy Caernarvonshire and Anglesey controls show no significant difference. Statistical tests show however, that this reversal is, in fact, not significant, and no obvious explanation of the reversal of the blood group relationship is apparent. Confirmation by further observations is required.

*Significance of pernicious anaemia morbidity*

The present survey is an assessment of pernicious anaemia morbidity, standardised for age, in two island communities. We consider that it is more accurate in its confined scope than nation-wide surveys based on death certification, or blood film examination, and related to practice populations or consultation rates per 1,000 population (Scott, 1960; Logan and Cushion, 1958; Payne, 1961). Mosbech's conclusion (1953) that there is a " common inherited predisposition " to stomach cancer and pernicious anaemia is consistent with the general regional association demonstrated elsewhere in England by these surveys (Wynne Griffith, 1960); but it certainly does not fit the findings so well in Anglesey. The incidence of pernicious anaemia, if not greater in the Isle of Man, is approximately the same in the two islands of Anglesey and Isle of Man, the observed number of pernicious anaemia patients in Anglesey being lower than expected.

*The alternative to heredity factors*

We conclude that if such factors as pernicious anaemia prevelance and blood group A association are related to the ordinary levels of incidence of stomach cancer, some other factor must also be related to the excessive incidence of stomach cancer in North Wales.

In other words we must account for it by postulating the existence of some environmental or exogenous agent. The exact nature of this agent is surely the secret of gastric cancer aetiology. Gorer (1938) points out that heredity and environment should not be regarded as alternative causes of cancer, but that heredity factors must be regarded as influencing the sensitivity of individuals to stimuli capable of giving rise to malignancy.

In the notable examples of chimney sweeps' scrotal cancer and the bladder cancer of aniline dye workers heredity factors presumably operate very weakly. And in the case of gastric cancer in North Wales where heredity factors are either overshadowed or at a low level of intensity we infer that a similar state of affairs exists. Specific carcinogenic agents have been isolated and chemically analysed in the case of scrotal and bladder tumours, but in the case of gastric tumours probably several factors combine to provoke malignant change. However, there seems to be sound justification for continuing the search for some specific gastric irritant or carcinogenic agent. Accumulated evidence points to the soil of certain parts of North Wales as the source of this agent.

### SUMMARY

Factors related to the aetiology of cancer of the stomach are reviewed, and in particular, genetic influences in that region of Britain where stomach cancer mortality is highest.

Data were obtained from the hospital records of 240 residents of Caernarvonshire and Anglesey who had died with a well-founded diagnosis of carcinoma of the stomach between the years 1952 and 1958. A sample of 68 of these was examined with respect to symptomatology and gastric acidity, and the total number with respect to anatomical site of the tumour and blood group where these had been recorded.

Carcinoma of the stomach in residents of Caernarvonshire and Anglesey shows no unexpected features in respect of symptomatology, sex distribution and gastric acidity. There is a high proportion of cases aged 70 years and over, predominantly female. A deficiency of juxta-pyloric carcinoma and an excess of lesser curve and body carcinomas are observed compared with the findings elsewhere in Britain and Europe.

Blood group determinations of 124 stomach cancer patients from Caernarvonshire and Anglesey do not reveal the expected increase of blood group A and deficit of blood group O compared with healthy controls in the two counties; in fact, there is a reversal of the usual association.

The incidence of pernicious anaemia in the populations at risk to it is not significantly different in Anglesey, an island of high stomach cancer mortality, and in the Isle of Man, an island of average stomach cancer mortality.

The four atypical features of gastric carcinoma in Caernarvonshire and Anglesey, namely, the unique blood group distribution, the unrelated pernicious anaemia incidence, the unusual distribution of site of origin of the tumours within the stomach and the high proportion of elderly cases, are all in conflict with principles of hereditary influence in the disease.

It is suggested that these peculiarities must be the result of specific environmental and exogenous factors powerful enough to obliterate evidence of the ordinary genetic factors. Such a hypothesis accords with the previous findings of a gastric carcinoma-soil relationship in this region.

I am indebted to Dr. Emyr Jones of the Caernarvonshire and Anglesey General Hospital, Bangor, who originally encouraged my interest in this work, and without whose assistance much of the data would not have been available.

Dr. J. S. Fulton and Dr. D. M. Fraser of the Liverpool Radium Institute and Dr. Gerald Evans of the Centre Pathology Laboratory, Bangor, kindly supplied me with information about hospital records of the patients.

I am also indebted to Dr. C. G. Pantin, pathologist of the Noble's Hospital, Douglas, Isle of Man, for his independent survey of pernicious anaemia in the island on my behalf, and to the general practitioners of Caernarvonshire and Anglesey for their co-operation in the survey.

The assistance of Miss Beryl Roberts, and the Records Officers at the Bangor, Llandudno and St. Asaph Hospitals and the Liverpool Royal Infirmary and the Royal Southern Hospital, Liverpool, is acknowledged.

Fig. 1 is reproduced by kind permission of Dr. P. Stocks and of Messrs. Butterworth & Co. Ltd.

### REFERENCES

AIRD, I. AND BENTALL, H. H.—(1953) *Br. med. J.*, i, 799.
BILLINGTON, B. P.—(1956) *Br. med. J.*, ii, 859.
COMFORT, M. W.—(1951) *Ann. intern. Med.*, **34**, 1331.
DAVIES, R. I. AND WYNNE GRIFFITH, G.—(1954) *Br. J. Cancer*, **8**, 56.
DENK, W.—(1940) *Z. Krebsforsch.*, **49**, 237.
GORER, P. A.—(1938) *Ann. Eugen.*, **8**, 219.
JENNINGS, D., BALME, R. R. AND RICHARDSON, J. E.—(1956) *Lancet*, i, 11.
KAPLAN, H. S. AND RIGLER, L. G. —(1947) *J. Lab. clin. Med.*, **32**, 644.
KØSTER, K. H., SINDRUP, E. AND SEELE, V.—(1955) *Lancet*, ii, 52.
LEGON, C. D.—(1952) *Br. med. J.*, ii, 700.
LOGAN, W. P. D. AND CUSHION, A. A.—(1958) 'Morbidity Statistics from General Practice', Vol. 1 (General). London (H.M. Stationery Office).
MOSBECH, J.—(1953) 'Heredity in Pernicious Anaemia'. Copenhagen (Munksgaard).
MOSBECH, J. AND VIDEBAEK, A.—(1950) *Br. med. J.*, ii, 390.
PAYNE, R. W.—(1961) *Br. med. J.*, i, 1807.
QUINCKE, ?.—(1876) Cited by Doehring, P. C. and Eusterman, G. B. (1942) *Archs Surg., Chicago*, **45**, 554.
SCOTT, E.—(1960) *J. Coll. gen. Practnrs Res. Newsl.*, **3**, 80.
STOCKS, P.—(1936) *Rep. Br. Emp. Cancer Campn*, **13**, 241.—(1947) 'Regional and Local Differences in Cancer Death Rates. Studies on Medical and Population Subjects, No. 1'. London (H.M. Stationery Office).—(1957) *Rep. Br. Emp. Cancer Campn*, **35**, Supplement to Part 2.—(1958) 'Cancer', edited by R. W. Raven, London (Butterworth), Vol. 3, p. 154.
SWYNNERTON, B. F. AND TRUELOVE, S. C.—(1952) *Br. med. J.*, i, 287.
TROMP, S. W. AND DIEHL, J. C.—(1955) *Br. J. Cancer*, **9**, 349.
VIDEBAEK, A. AND MOSBECH, J.—(1954) *Acta med. scand.*, **149**, 137.
VERSCHNER, O. AND KOBOR, E.—(1940) *Z. Krebsforsch.*, **50**, 5.
WYNNE GRIFFITH, G.—(1960) *Med. Press*, **244**, 93.